# New Biofuel Integrating Glycerol into Its Composition Through the Use of Covalent Immobilized Pig Pancreatic Lipase

**DOI:** 10.3390/ijms130810091

**Published:** 2012-08-13

**Authors:** Diego Luna, Alejandro Posadillo, Verónica Caballero, Cristóbal Verdugo, Felipa M. Bautista, Antonio A. Romero, Enrique D. Sancho, Carlos Luna, Juan Calero

**Affiliations:** 1 Department of Organic Chemistry, University of Cordoba, Campus de Rabanales, Ed. Marie Curie, 14014, Córdoba, Spain; E-Mails: vcmvanadio@gmail.com (V.C.); qo1baruf@uco.es (F.M.B.); qo1rorea@uco.es (A.A.R.); qo2luduc@uco.es (C.L.); p72camaj@uco.es (J.C.); 2 Seneca Green Catalyst S.L., Campus de Rabanales, 14014, Córdoba, Spain; E-Mail: seneca@uco.es; 3 Crystallographic Studies Laboratory, Andalusian Institute of Earth Sciences, CSIC, Avda. Las Palmeras n°4, 18100, Armilla, Granada, Spain; E-Mail: cverdugoe@lec.csic.es; 4 Department of Microbiology, University of Córdoba, Campus de Rabanales, Ed. Marie Curie, 14014, Córdoba, Spain; E-Mail: mi1sapue@uco.es

**Keywords:** biofuels, sunflower oil transesterification, covalent immobilization, pig pancreatic lipase (PPL), Sepiolite, amorphous AlPO_4,_ monoglyceride

## Abstract

By using 1,3-specific Pig Pancreatic lipase (EC 3.1.1.3 or PPL), covalently immobilized on AlPO_4_/Sepiolite support as biocatalyst, a new second-generation biodiesel was obtained in the transesterification reaction of sunflower oil with ethanol and other alcohols of low molecular weight. The resulting biofuel is composed of fatty acid ethyl esters and monoglycerides (FAEE/MG) blended in a molar relation 2/1. This novel product, which integrates glycerol as monoacylglycerols (MG) into the biofuel composition, has similar physicochemical properties compared to those of conventional biodiesel and also avoids the removal step of this by-product. The biocatalyst was found to be strongly fixed to the inorganic support (75%). Nevertheless, the efficiency of the immobilized enzyme was reduced to half (49.1%) compared to that of the free PPL. The immobilized enzyme showed a remarkable stability as well as a great reusability (more than 40 successive reuses) without a significant loss of its initial catalytic activity. Immobilized and free enzymes exhibited different reaction mechanisms, according to the different results in the Arrhenius parameters (Ln A and Ea). However, the use of supported PPL was found to be very suitable for the repetitive production of biofuel due to its facile recyclability from the reaction mixture.

## 1. Introduction

The lipid fraction of biomass has been identified as carbon neutral substitution of fuels from fossil sources in the transportation sector. Although the diesel engine, invented by Rudolph Diesel over a century ago, first ran on peanut oil, the current combustion engines are designed to run on hydrocarbon fuels derived from petroleum. Therefore, a substitute for diesel fuel from renewable source will need to have identical or closely similar properties. The most popular of the existing technologies for processing vegetable or animal oils is based on the conversion of the triglycerides constituents to fatty acid methyl esters (FAME). Other alternative processing routes are dilution of the vegetable oils, emulsification, pyrolysis and hydrotreating [1]. There are several methods under development to remove glycerol without using water. The production of glycerin as a byproduct is a serious problem with the current conventional technology in FAME fabrication because in most cases it is necessary to use a lot of water for the removal of the residue of glycerol in biodiesel before its use. In fact, this drawback is actually also the main difficulty associated with the fabrication of biofuels in many Mediterranean countries and other worldwide regions where water is not abundant.

Current industrial production of *biodiesel* (“mono alkyl esters of long chain fatty acids derived from renewable lipid feedstock, such as vegetable oils or animal fats, for use in compression diesel engines”—ASTM definition) is carried out by homogeneous alkali-catalyzed transesterification of vegetable oils with methanol, using sodium hydroxide, potassium hydroxide or potassium methoxide as catalysts [2]. The homogeneous basic transesterification reaction shows a very fast kinetic rate, but unfortunately, a collateral saponification reaction takes place, reducing the biodiesel production efficiency. To prevent the biodiesel yield loss due to the acid-base reaction, an acid pre-treatment is often needed for vegetable oils having more than a 5 wt.% of free fatty acid (FFA) in order to improve the biodiesel efficiency production [1–4]. Biodiesel is finally recovered by repeated washing with water to remove glycerol, soap and excess of methanol [1–3]. In contrast, the acid transesterification allows obtaining a biodiesel production without formation of by-products. Drawbacks of an acid homogeneous transesterification include the use of corrosive catalysts (H_2_SO_4_, H_3_PO_4_, HCl) and slow reaction rates. These may be increased at high temperatures and pressures, involving larger costs [2]. However several reports can be recently found on the production of biodiesel involving other chemicals [4,5], the use of heterogeneous base catalysts [6–8], solid acids [9–11] or bifunctional solids (acid–base character) which show an ability to simultaneously catalyze esterification and transesterification reactions [12] as well as enzymatic protocols as greener alternatives [13–16].

Regardless of the procedure for obtaining the conventional biodiesel, glycerin is obtained as a byproduct in all cases, representing a notable performance loss of the process. The achievement of new biofuels that integrate the glycerin as an alternative product miscible with FAME is currently a target of great interest, given that the market is already virtually flooded by the production of glycerine, precisely obtained as a by-product in the current manufacturing of biodiesel [17,18]. These biofuels not only prevent the generation of waste but are also found in increased yields during the process, which are always higher than nominal 10%, by incorporating some derivative of glycerine in the reaction products. Novel methodologies to prepare esters from lipids using different acyl acceptors, which directly afford alternative co-products are currently under development [13,19–21]. The transesterification reaction of triglycerides with dimethyl carbonate (DMC) [22–26], methyl acetate [27–31] or ethyl acetate [32,33] can generate a mixture of three molecules of FAME or FAEE and one of glycerol carbonate (GC) or glycerol triacetate (triacetin). These mixtures including glycerine derivative molecules have relevant physical properties to be employed as novel biofuels [34,35]. The atom efficiency is also improved, as the total number of atoms involved in the reaction is part of the final mixture.

In this context, we have recently developed an alternative protocol for the preparation of novel biofuels integrating glycerol into their composition via 1,3-regiospecific enzymatic alcoholysis of sunflower oil using some “1,3” regioespecific low cost lipase, like pig pancreatic lipase, PPL [36–38] or from *Thermomyces lanuginosus* (Lipopan 50 BG from Novozymes) [39], As in the two methods described in [19–35], the already patented Ecodiesel-100 [38] obtained through the 1,3-selective partial ethanolysis of the triglycerides with PPL, is a mixture of two parts of FAEE and one part of MG, that integrates the glycerine as a soluble derivative product (MG) in diesel fuel, but unlike these methods, no specific reagent (such as dimethyl carbonate or methyl acetate) more expensive than methanol (or ethanol) is used. This strategy is based on obtaining an incomplete alcoholysis by application of 1.3 selective lipases, so that the glycerol remains in the form of monoglyceride, which avoids the production of glycerol as by-product, reducing the environmental impact of the process. Thus, the operating conditions compared to the conventional method are much smoother, no impurities need to be removed from the final mixture and the biofuel obtained exhibits similar physical properties to those of conventional biodiesel [36–39]. In this respect, some studies have also demonstrated that minor components of biodiesel, usually considered contaminants under the biodiesel standard EN 14214, including free fatty acids and monoacyl glycerol, are essentially responsible for the lubricity of low-levels blends of biodiesel and petrodiesel. Pure FAME exhibit a reduced lubricity compared to the biodiesel containing these compounds [40–44]. The presence of greater quantities of monoglycerides and/or free fatty acids enhances the lubricity of biodiesel, which is another key feature of this novel biofuel that incorporates high amounts of monoglycerides.

The actual existing limitations for the use of industrial lipases have been mainly associated with their high production costs, which can be overcome through the application of molecular technologies to achieve the production of enzymes purified in sufficiently high quantities as well as by the reuse of biocatalysts after the heterogeneous immobilization of lipases [45,46]. Here, we report the application of an enzymatic protocol using free and covalent immobilized pig pancreatic lipase (PPL) to obtain the “1,3” selective alcoholysis of sunflower oil with ethanol and other short chain alcohols. In this respect, covalent immobilization of PPL is carried out by using a methodology previously described in the heterogeneization of lipases [47–50], other enzymes like phosphatase [51] or glucose oxidase [52] as well as homogeneous organometallic complex [53,54]. Taking into account the excellent results obtained in the immobilization of these enzymes, in this study we now have extended the possibilities of this methodology to be applied to the covalent attachment of PPL to be applied as an economically viable biocatalyst for the production of a novel biofuel integrating glycerine into its composition.

## 2. Results and Discussion

In this study, the kinetic properties of PPL were obtained when covalently immobilized on amorphous AlPO_4_/clay mineral Sepiolite system as inorganic supports. Enzymatic activities of native and immobilized PPL were determined under different working conditions such as reaction temperature, reaction pH, oil/alcohol ratio and enzyme concentration.

**Figure 1:**
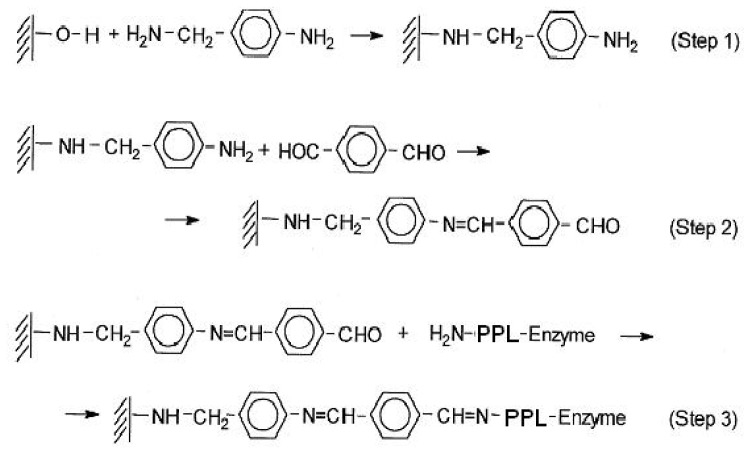
Immobilization of Pig Pancreatic lipase (PPL) through the ɛ-amino group of lysine residues to an organic linker bonded to the inorganic support. (**a**) In Step 1, surface OH groups of the supported AlPO_4_/Sepiolite are activated by microwave heating with 4-aminobenzylamine; (**b**) In Step 2, tereftaldicarboxaldehyde is reacted through imines bonds also obtained by microwave heating; (**c**) In Step 3 the covalent immobilization of the enzyme is obtained through the imines bonds produced with lysine residues.

AlPO_4_/Sepiolite (20–80% weight) was used as support for the covalent immobilization of PPL after its functionalization, according to the solid phase stepwise synthesis in Figure 1. Functionalization of the support surfaces is developed by anchoring a functionalized linker with pendant benzaldehyde through the reaction of appropriate molecules with the surface –OH groups of inorganic solids. Thus, an organic linker is built through the reaction of surface OH groups of the supported AlPO_4_/Sepiolite with 4-aminobenzylamine (Step 1), followed by its reaction with tereftaldicarboxaldehyde (Step 2). Covalent immobilization of the enzyme is carried out through the ɛ-amino group of lysine residues (Step 3) in agreement with previous reports [47–52].

**Figure 2:**
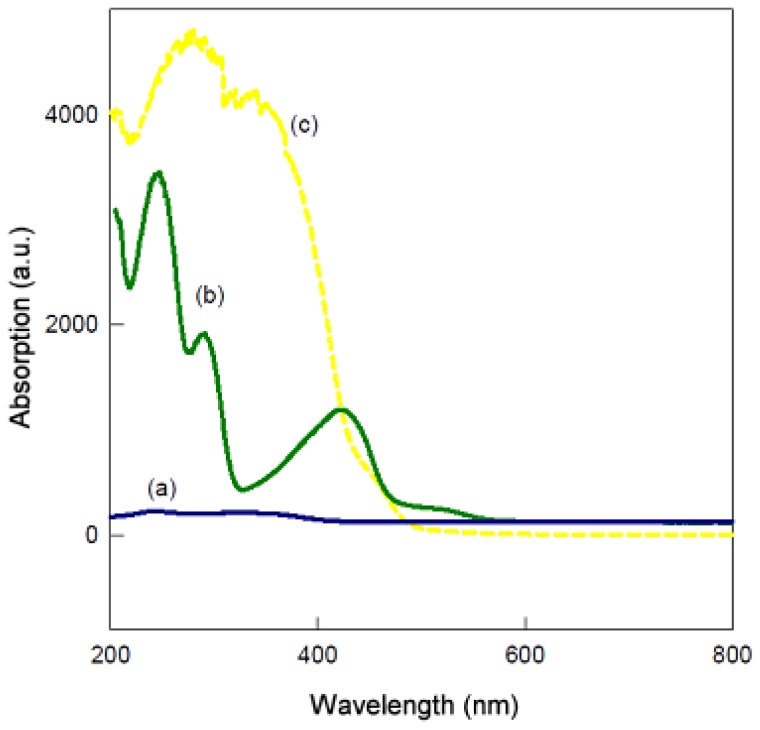
Visible-Ultraviolet Diffuse Reflectance spectra of different samples obtained along different steps in Figure 1. (**a**) Sepiolita/AlPO_4_ support; (**b**) activated support with 4-aminobenzylamine after microwave heating; (**c**) Functionalized support after reaction of activated support with tereftaldialdehyde under microwave heating.

The activation of AlPO4 surfaces (supported on Sepiolite) was carried out through the formation of phosphamide bonds, obtained by the reaction of the Brönsted acid sites on the support surfaces, with 4-aminobenzylamine. In every case, the proper functionalization was obtained in a next step, by the chemical modification of the pendant aniline molecule, obtained after the reaction of the benzylamine group with the surface acid –OH groups of inorganic solids. The efficiency of the immobilization process is confirmed by UV-vis-DR that checks the changes in the surface of the inorganic solid used as support in the successive treatments that lead to the functionalization with superficial aldehyde groups, which are able to immobilize enzymes by the reaction with ɛ-lysine of proteins, Figure 2.

The functionalization of Sepiolite, used as inorganic support is possible due to the presence of a large number of surface acid hydroxyl groups in AlPO_4_, used as inorganic activating material. The activation of AlPO_4_ surfaces is carried out through the formation of phosphamide or phosohoester bonds, obtained by the reaction of the Brönsted acid sites on the support surfaces, with 4-aminobenzylamine (or another diamine). In every case, the proper functionalization that leads to enzyme inmobilization is obtained in a next step by the chemical modification of the pendant aniline molecule, after the reaction with a tereftaldicarboxaldehyde (or another dialdehyde), so that the activation process is carried out in two steps, making it possible to be performed in the same reaction flask, after washing the solid after each reaction. Moreover, both reactions are done in a quick and clean way in a conventional microwave. The amorphous material used as support is tailored by a controlled sol–gel method that allows us to obtain a high surface area as well as a high number of surfaces –OH groups, and is a very adequate support component for the covalent attachment of enzymes. Due to the increased electrophilic character of benzaldehyde, the aromatic Schiff’s-base obtained seems to be more stable than those usually obtained by using glutaraldehyde [55,56] or other aliphatic aldehydes like glycidol [55–57].

Covalent binding to an insoluble support is the most interesting enzyme immobilization methodology because it combines the high selectivity of enzymatic reactions with the chemical and mechanical properties of the support [55–58]. Covalent immobilization on the external surface of a support material has also been proposed to decrease mass transfer limitations associated with several immobilization techniques, such as entrapment or adsorption in gels. The majority of the research in this area has been focused on polymer-supported carriers because of the presence of many different functional groups, which can provide efficient interactions with the enzymes. However, the organic supports suffer a number of problems such as a significant loss in the stability of the catalytic material throughout the successive uses, mainly due to its swelling and poor stability towards microbial attacks and organic solvents.

Comparatively, the main advantages of inorganic supports are their great physical strength and chemical inertness. Thus, some materials such as silica, alumina, and layered double hydroxides are known to be thermally and mechanically stable. Besides, inorganic matrices have a number of advantages over organic ones: No swelling and no porosity changes occur with pH; there is excellent storage stability of enzymes, and they are not subject to microbial attack, so that the immobilization onto inorganic supports could thus improve the catalytic performance [58–61].

In most of the studies reported to date on covalent immobilization on inorganic supports, amorphous or mesoporous silica has generally been used as the support. Micelle template silica constitutes in fact a new class of materials with high thermal and mechanical stability, which can be modified and functionalized, either during its synthesis by the sol-gel method, or by directly grafting the functional organosilane groups on silica surfaces [61–63]. In the second case, the catalyst stability always depends on the stability of the different organosilane bonds, which can be broken due to effects of the reaction conditions. Thus, water solutions, polar solvents, higher temperatures, *etc*., may promote the hydrolysis of the organic–inorganic hybrid bonds, thus leading to varying degrees of metal leaching.

In order to overcome this drawback, in this study we try to improve this methodology using organic–inorganic hybrid bonds, which are more stable and hydrolysis-resistant than the organosilane bonds. Thus, here we explore the possibilities of the phosphamide bond to get more efficient links between different organic molecules and the external surface of an amorphous inorganic solid such as AlPO_4_. Thus, the high stability of phosphamide and phosphoester bonds plays a crucial role in the well-established structure of many different biomolecules, including RNA and DNA macromolecules.

In this regard, it is well known that under the common name of zeotypes, we identify a family of crystalline oxides built up from corner-shared *T*O_4_ tetrahedra (*T* = Si, Al, Ga, P) whose structure contains internal cavities and channels of variable shape and size. Thus, the original family of silica-based zeolites and aluminosilicates (SAPOs) has been greatly extended in recent decades, to include aluminophosphates (AlPOs), that, since their discovery by scientists at the Union Carbide Corp. in the early 1980s [64], constitute a wide family of new materials that are among the most studied and most frequently employed as catalysts as well as supports in several organic reactions. In general, they all exhibit high thermal and mechanical stability and can be deeply modified throughout their synthesis by hydrothermal and sol-gel methods, thus obtaining a large amount of crystalline materials ranging from microporous to mesoporous structures [65].

Furthermore, amorphous AlPO_4_ has attracted the interest of many researchers due to the realization that AlPO_4_ is completely iso-structural with silica and it exhibits parallel polymorphic transformation [66], although the reasons for its high catalytic activity remain a point of contention. Thus, in the last few years we have undertaken a series of research studies on the synthesis, characterization and catalytic activity of different amorphous AlPO_4_, in which textural and acid–base properties were dependent on a number of variables such as aluminum starting salt, P/Al ratio, precipitation medium, or thermal treatment during drying and calcinations [67].

According to the results obtained in this work this amorphous AlPO_4_ material, properly tailored by a controlled sol–gel method that allows obtaining a high surface area as well as a high number of surface Brönsted acid sites, has been a very adequate component for the covalent attachment of PPL. Sunflower oil is composed of a mixture of fatty acids (mainly oleic, linoleic and stearic acids) in varying proportions. The effect of the different parameters on the ethanolysis of sunflower oil using free PPL has been investigated in recent papers in order to optimize the reaction conditions [36–38]. In this way, reaction tests of PPL covalently supported in AlPO_4_/Sepiolite systems have been carried out under the optimum conditions (pH environments, temperatures and relative oil/ethanol ratio) determined for free PPL. In this respect, Tables 1–4 show a collection of the results achieved in the transesterification reaction with a unique biocatalyst, constituted by immobilized PPL on AlPO_4_/Sepiolite as support. Thus, in addition to obtaining specific information about the influence of certain parameters (pH temperature *etc*.) on the behavior of the immobilized PPL, their ability to be reused may be assessed.

On the other hand, from the results in Table 1 we can determine the amount of enzyme covalently immobilized on the activated surface of the support in the immobilization process, see Step 3 Figure 1. In this sense, based on the differences in enzymatic activity between the supernatant PPL, removed by washing (6 mL of ethanol) in the immobilization process (Table 1, No. 0b), and those prepared in free form (Table 1, No. 0a), operating under the same experimental conditions, the amount of covalently immobilized enzyme can be determined because the enzyme activity is normally proportional to the amount of enzyme in solution. Thus, the amount of PPL enzyme supernatant resulting from this calculation is 0.009 g. Therefore, the amount of enzyme which is immobilized is the difference between the introduced and the supernatant remaining free enzyme, 0.04 – 0.009 g = 0.031 g. That is, 77.5% of covalent immobilization of the PPL employed in the immobilization process is obtained. The covalent immobilization achieved falls within the limits obtained with other immobilized enzymes using this methodology [51,52]. Likewise, knowing the amount of immobilized PPL makes it possible to obtain the corresponding Turn Over Frequencies (TOF, mol h^−1^ g_PPL_^−1^), reaction rates values calculated from the FAEE yield per unit of reaction time and weight of PPL employed.

From the comparison of the activity of PPL in free form with respect to the immobilized enzyme, both determined under the same experimental conditions and temperature, the efficiency of the PPL after its covalent immobilization is obtained. Thus, by comparing the TOF values of free PPL, 4.75 (Table 1, No. 0a) and immobilized, 2.33, (Table 1, No. 6), the efficiency of immobilized PPL (2.33/4.75) × 100 = 49.05% is determined. This is a very informative parameter because it is able to define an extensive, but ineffective immobilization, because the enzyme was deactivated after immobilization. This deactivation may be due, among other causes, to the involvement of one or more lysine amino acids of the active sites of enzymes in the bond to the support. From the results collected in Table 1, it can also be concluded that like the enzyme in free form, the immobilized form experiences a remarkable influence of the pH in the transesterification reaction with ethanol, although with less intensity than the free form [37].

The study of the influence of the oil/alcohol ratio on the activity of the immobilized PPL was also carried out with the same biocatalyst previously used 14 times, as described in Table 2, numbered in a sequential form, so that the reuse of the immobilized enzyme continued. The results obtained indicate that, as it is described with the free PPL enzyme [37], and contrary to what happens in a homogeneous process with basic catalysts NaOH or KOH, the ethanolysis of sunflower oil is not increased with increasing amounts of alcohol in respect to the vegetable oil amount.

The same covalently immobilized PPL biocatalyst can still be repeatedly used and the results are shown in Tables 3 and 4. In the former there is a collection of a series of transesterification reactions with methanol under two different pH values where varying amounts of glycerin (GLY) are obtained. The latter one presents results obtained operating with 1-propanol and 2-propanol. The results obtained when 1-propanol and 2-propanol were used in the transesterification reaction clearly show the sensitivity of the immobilized PPL with respect to the structure of the alcohols. Thus, as it can be seen in the results in Table 4, the primary alcohol develops the alcoholysis process more easily than the secondary alcohol at all the different temperatures studied, operating at pH = 10, a volumetric ratio oil/alcohol 2/1 and under standard conditions.

On the other hand, according to the results previously described with the PPL in free form [37] Table 5, and those listed in Tables 1–4, it can be seen that this enzyme seems to show less influence in respect to the alcohol used in the transesterification process when it acts in the free form than when it operates covalently immobilized. In this way, although PPL in the free form has a very similar behavior with different alcohols without marked differences between them [37] (Table 5), with the immobilized PPL, according to the results in Tables 1 and 3, methanol seems to present a higher catalytic activity especially at pH = 10, where the production of glycerin is also obtained.

Indeed, the most notable difference between the results shown in Tables 1 and 3 is that it appears that ethanol, at pH = 10, presents a greater barrier for the alcoholysis of MG that produces a molecule of glycerin and a third molecule of FAEE, than the methanol, that leads to glycerin and the corresponding FAME. Consequently, ethanol will be a more adequate alcohol than methanol to be used in a transesterification oil process to obtain a biofuel that integrates glycerin.

We can see that the PPL enzyme presents a higher enzyme activity when it is free, as well as that the variations found with immobilized PPL under the various operating conditions, including pH and/or alcohol, acquire a single relative importance only with ethanol. In other cases there are no significant variations among alcohols, with the exception of the differences between primary and secondary alcohols like 1-propanol and 2-propanol where the sensitivity of the immobilized PPL with respect to the structure of the alcohols is clearly shown. Thus, according to the results, the primary alcohol develops the alcoholysis process more easily than the secondary alcohol.

These results are in agreement with those in the literature concluding that ethanolysis is the best option to improve the enzymatic biodiesel synthesis as compared to other alcohols, including methanol [68–70]. It is also interesting to note that no selectivity values higher than 60%–70% were found in any case, independent of the used alcohol. This is easily explained by the 1,3 regiospecific alcoholysis of sunflower oil with ethanol and other short chain alcohols previously described using free PPL [37].

Lipases have indeed a peculiar 1,3-regioselectivity, which means that they selectively hydrolyse the more reactive 1 and 3 positions in the triglyceride [71]. In this regard, the production of biodiesel using lipases needs to take into account this regiospecific character [72,73] responsible for obtaining selectivities in FAME or FAEE lower than 70 wt.% in the alcoholysis of triglycerides [74,75]. Thus, the enzyme catalyzed biofuel production does not generate any glycerine as a result of the 1,3 selective hydrolysis of the triglycerides in the ethanolysis of sunflower oil. A potentially useful biofuel blend of FAEE, MG and traces of DG, in varying proportions (depending on the conversions) was obtained. Results also pointed out that, even with an excess of ethanol, a maximum 66% yield could be obtained in every case, corresponding to a 1,3 selective enzymatic process.

On the other hand, this biofuel that integrates glycerol as the monoglyceride not only improves the efficiency of the process but also presents physico-chemical properties very similar to conventional biodiesel obtained by alkaline catalysis, as can be seen in Table 6, where they all exhibited viscosity values similar to commercial diesel 3.1 cSt., when methanol or ethanol are used in the alcoholysis process. Here we can see how the viscosity is indeed highly dependent on the proportion of the TG in the sunflower oil (given its high viscosity value, 31.9 cSt), and, to a lesser extent, on the mixture MG + DG (only traces of DG could be found at FAEE conversions above 50%). The presence of MG was also expected to have little influence on the viscosity of the biofuel. In any case, by blending these biofuels with diesel in a B20, the viscosity values are reduced enough to fulfill the required reference (3.0–5.0 cSt). Thus, a B20 obtained from a 20% blend with commercial diesel, using the worst biofuel (υ = 12.9 cSt) showed a 4.2 cSt value.

**Figure 3:**
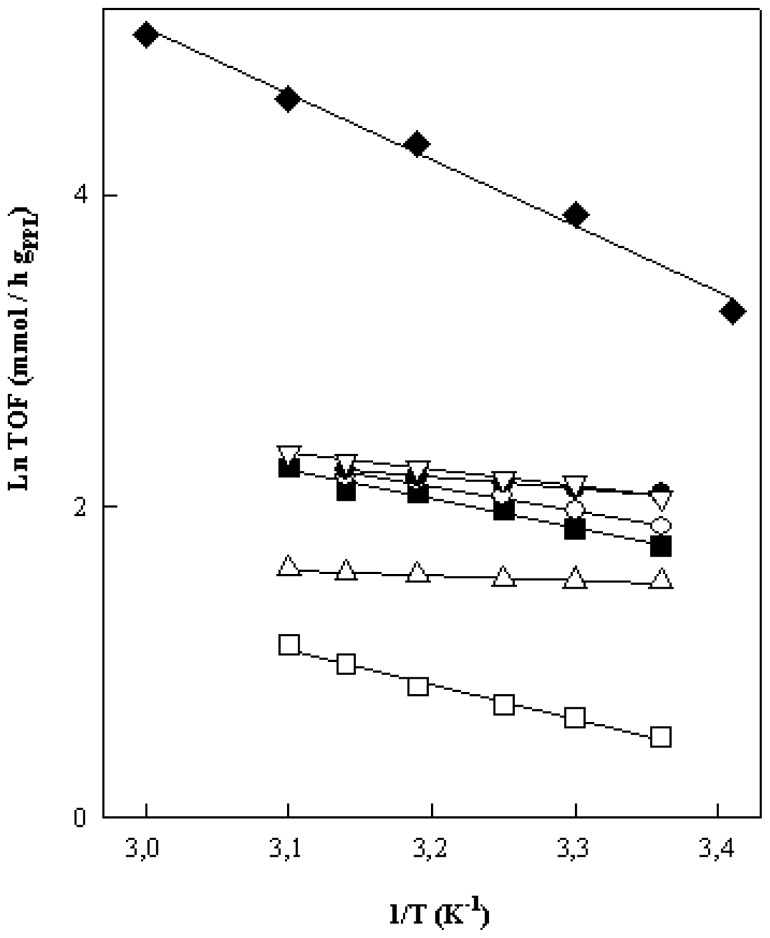
Arrhenius plots (Ln TOF *vs*. 1/*T*) comparing the enzymatic activities of free PPL at pH = 12 (♦) with immobilized *PPL*, using different alcohols and pH values: (□) EtOH, pH 10; (■) EtOH, pH 12; (○) MetOH, pH 10; (●) MetOH, pH 12; (Δ) 1-PrOH, pH 10; (△) 2-PrOH pH 12.

Some additional information in respect to the differential mechanisms of enzymatic catalysis of PPL, operating under the different studied experimental conditions (free and immobilized, with various alcohols at two pH values) can be obtained from the results collected in Tables 1–4 of the different studied process at different temperatures. Thus, the representation of Arrhenius, according to Figure 3, can be obtained, which allows us to calculate the activation energies and Arrhenius constants from the slopes and intercepts, respectively, which are collected in Table 7.

In this respect, the numerical values of *E*_a_ and Ln A collected in Table 5 quantify in some way the effect of the immobilization and experimental conditions on the efficiency of PPL, because *E*_a_ provides an insight into the efficiency of the enzyme active sites whilst the Arrhenius constant, Ln A, gives information on the number of active sites able to intervene in the enzymatic process. Besides, the similarity of both values in the two processes manifests the existence of a common reaction mechanism. In this respect we have that the numerical values of *E*_a_ and Ln A (Table 7) that are very dissimilar between the free and immobilized enzyme, so that the free and covalently immobilized PPL must operate with different reaction mechanisms. The influence of pH as well as the structure of the alcohols is also detected in these kinetic parameters.

The comparison between the values of both parameters (*E*_a_ and Ln A) with those of the free enzyme shows that after the covalent immobilization of enzymes PPL, both a reduction in the relative number of active catalyst centers capable of participating in the process as well as a lesser activity of each enzyme immobilized in the reaction and/or the deactivation of the active sites of the enzyme in the entrapment process is obtained, which may be due to a steric effect in the covalent immobilization of the enzymes. The length of the organic linker used in the immobilization process could play an important role in this respect.

In summary, we found that, although the efficiency of immobilized PPL was reduced compared to the free form, its covalent immobilization guaranteed the lifespan of the lipases. Thus, while free PPL was completely deactivated in 48 h, the immobilized enzyme was maintained active for several weeks, even after successive reuses (in Tables 1–4 the results of 43 successive reactions are shown), preserving over 90% of the initial activity.

However, the life of these immobilized PPL is expanded in an extraordinary way from no more than 48 h with free PPL to months with successive reuse (in Tables 1–4 the results of 43 successive reactions are shown), maintaining a practically constant activity. Such a high number of reuses is not usually described in the literature with polymer organic supports or with inorganic supports activated with organosilane linkers [55–63,69,76].

## 3. Experimental Section

### 3.1. Support Synthesis and Support Functionalization

An AlPO_4_/Sepiolite system (20–80 wt.%) was used as support. The Sepiolite, a natural hydrated magnesium silicate of fibrous nature, with a high surface area and low price, was subjected to a surface activation process, through a soft precipitation as a gel of AlPO_4_ to obtain a final relationship Sepiolita/AlPO_4_ 80/20. It was obtained by adding sepiolite to a reaction medium where the precipitation of AlPO_4_ was initiated. The synthesis of AlPO_4_ was carried out according to a sol–gel method previously described [47–52]. This amorphous material was obtained by precipitation from aqueous solutions of AlCl_3_·6H_2_O and H_3_PO_4_ (85 wt.%). at pH = 6.1 by addition of ammonium hydroxide. The solid obtained after filtration was then washed with isopropyl alcohol and dried at 120 °C for 24 h. In the present case, the resulting powder was calcined by heating at 350 °C for 3 h and then screened to a particle size <0.149 mm (100 mesh size). The sepiolite (Tolsa S.A, Spain) is a natural silicate that presents a fibrous structure (Figure 1). The theoretical formula of the unit cell is Si_12_O_30_Mg_8_(OH)_6_(H_2_O)_4_·8H_2_O, where the Si^4+^ and the Mg^2+^ can be partially substituted by Al^3+^, Fe^2+^ and alkaline ions. Each atom of Mg completes its coordination with two molecules of water.

Activation of the AlPO_4_ support surface was carried out according to the general methodology outlined in Figure 1. In this way, the Sepiolite incorporated a thin outer layer of AlPO_4_, which made it acquire the physical-chemical properties of this compound, which later would allow the functionalization by reaction with an organic molecule. This process was initiated by anchoring a functionalized linker through the reaction of 4-aminobenzylamine with support surface –OH groups [47–52] according to Step 1 (Figure 1). The linker was obtained by a microwave heating reaction (15 min at 380 W) of the support (20 g) and 4-aminobenzylamine (4 g) and, after that, the composite was reacted with terephthaldicarboxaldehyde (Step 2) by microwave heating (5 min at 380 W). As a consequence of the high conjugation of the molecule, a yellow solid was obtained. The activation process was carried out, therefore, in two steps possible to perform in the same reaction flask, washing the solid after each reaction. Moreover, both reactions were done in a quick and clean way in a conventional microwave. The efficiency immobilization of the process was confirmed by Visible-Ultraviolet/Diffuse Reflectance, on a UV-Visible Diffuse Reflectance SpectrophotometerVarian Carey 1E, between wavelengths 200 and 900 nm. We checked the changes in the surface of the inorganic solid used as support in the successive treatments that lead to the functionalization with superficial aldehyde groups able to react and immobilize the enzyme’s proteins (Figure 2).

### 3.2. PPL Immobilization and Enzymatic Activity

The immobilization of PPL was carried out at room temperature by introducing the functionalized inorganic solid (0.5 g) with the PPL (0.04 g) in a reaction flask (50 mL) with 6 mL of ethanol, soft mechanical stirring and refrigerating for 24 h, stirring occasionally every three to four hours to get the covalent interaction of the ɛ-amino group of lysine residues of the PPL with the aldehyde groups of linkers (step 3). Finally, prior to its use, 6 mL ethanol was added to the mixture and the solid, with the immobilized PPL, was then separated by filtration and centrifugation from the solution containing the remaining non-immobilized lipase. The catalytic activity of this dissolution was proportional to the amount of PPL dissolved. Thus, we could easily determine the quantity of PPL, which had not been immobilized, and was hence remaining in supernatant dissolution. The comparison of this value with the activity of immobilized and free PPL enzymes would allow us to determine the amount of immobilized enzyme and its efficiency [47–52].

### 3.3. Alcoholysis Reactions

The alcoholysis reactions were performed in a 50 mL round bottom flask under continuous magnetic stirring at controlled temperature (25–50 °C) varying the pH values in the 8–12 range. The various pH environments were achieved by adding different quantities of aqueous solutions of NaOH 10 N. The reaction mixture was comprised of 9.4 g (12 mL, 0.01 mol) sunflower oil, a variable oil/alcohol volume ratio and 0.5 g of solid containing 0.01 g immobilized PPL. Free PPL (0.01 g) was also used as reference to determine the efficiency and amount of immobilized enzyme.

A commercial crude PPL (Type II, L3126, SIGMA-ALDRICH, USA), sunflower oil for food use and several short-chain alcohols (methanol, ethanol, 1-propanol, 2-propanol (Panreac, 99%)) were employed in the enzymatic alcoholysis reactions. Ethanol from Alcoholes del Sur, 96% was also used in this respect.

#### 3.3.1. Compositional Analysis of Reaction Products by Gas Chromatography

Samples were periodically withdrawn at different reaction times (6–48 h) and quantified using a gas chromatograph HP 5890 Series II Gas connected to a capillary column HT5, 0.1 UM (25 m × 0.32 mm, SGE). Dodecane was employed as internal standard. The results are expressed as relative quantities of the corresponding Fatty Acid Esters (FAE) and the sum of the quantities of MG and diglycerides (DG). As FAE may be considered FAME, FAEE *etc*., the yield refers to the relative amount of FAEE produced (%). The conversion includes the total amount (%) of triglyceride transformed (FAE + MG + DG). The reaction rates and turn over frequencies (TOF, mol h^−1^ g_PPL_^−1^) were calculated from the yield, considering the amount of every FAE generated per unit of time of reaction and weight of PPL employed. The blank reaction in the presence of the highest quantity of solution of NaOH was performed to rule out a potential contribution from the homogeneous NaOH catalyzed reaction. Less than 15% conversion of the starting material was found under this condition implying that the production of the biofuel was carried out by the enzyme added as catalyst.

#### 3.3.2. Viscosity Measurements

The viscosity was determined in a capillary viscometer Oswald Proton Cannon-Fenske Routine Viscometer 33,200, size 200. This is based on determining the time needed for a given volume of fluid passing between two points marked on the instrument. It correlates to the speed reduction suffered by the flow of liquid as a result of internal friction of its molecules, depending on their viscosity. From the flow time, *t*, in seconds, the kinematic viscosity (*υ*, centistokes, cSt) can be obtained from the equation: *υ* × *t* = *C*, where *C* is the constant calibration of the measuring system in cSt·s, which is given by the manufacturer (0.10698 mm^2^ s^−1^, at 40 °C) and t the flow time in seconds. The kinematic viscosity also represents the ratio between the dynamic viscosity and the density (*ρ*, *υ* =*η*/*ρ*).

## 4. Conclusions

The alcoholysis of sunflower oil with several short-chain alcohols using covalent immobilized 1,3-regiospecific PPL can play an advantageous role compared to the conventional base catalyzed processes to prepare new biofuels incorporating glycerin as monoglycerides, obtaining in this way a reduction in the production of waste, an improvement in the reaction conversion (100% theoretical) and greener operating conditions. The atom efficiency is also improved to 100% because the total number of atoms involved in the reaction (as reactants) becomes part of the final mixture that constitutes the biofuel. This new biofuel is out of the standard EN 14214, however several papers have demonstrated that monoglycerides enhance the lubricity of fuels in respect to standard biodiesel [40–44], so that its incorporation into the market of biofuels currently has no technical limitations.

In this respect, the results obtained open the possibility to use different alcohols than methanol, and they expand the possibility to use not only PPL but also every “1,3” selective lipase that currently is only able to easily obtain 66%–70% conversion due to the difficulty in obtaining the alcoholysis of the 2-fatty acid esters of glycerol, due to the fact that they are only able to selectively hydrolyse the more reactive 1 and 3 positions in the triglyceride. In this regard, the production of conventional biodiesel using lipases needs to take into account such regiospecific characteristics responsible for comparatively long reaction times and conversions lower than 70 wt.% in fatty acid methyl or ethyl esters [74,75]. However this weakness becomes a strength when we consider an alternative objective consisting of obtaining a biofuel that integrates glycerol as the monoglyceride, because it presents physico-chemical properties similar or even better than conventional biodiesel as well as all kinds of benefits related to the quality of the biofuel and the fabrication process.

On the other hand, it was found that, following the studied methodology, PPL was strongly fixed to the inorganic support (75%). Nevertheless, the efficiency of the immobilized enzyme was reduced to half (49.1%) compared to that of the free PPL, but the covalently immobilized enzymes showed a remarkable stability as well as a great reusability (more than 40 successive reuses) without a significant loss of their initial catalytic activity. This indicates the capability of the phosphamide bond to create efficient links between different organic molecules and the external surface of an amorphous inorganic solid such as AlPO_4_. Thus, the high stability of phosphamide bonds plays a crucial role in the covalent immobilization of PPL biomolecules. Consequently, based on the phosphamide bond, a versatile procedure is achieved to obtain hybrid organic–inorganic molecules able to produce the covalent attachment to inorganic supports of many different organic molecules, including biomolecules like lipase enzymes. Thus, within the same general schemes currently used with silanized SiO_2_ [77–80], these organic molecules can by modified following the experimental methodology of the solid-phase synthesis, to obtain adequate ligands linked to solid surfaces.

## Figures and Tables

**Table 1:** Composition, yield, conversion (Conv.) and Turn Over Frequencies (TOF) of the biodiesel obtained in the transesterification reaction of 0.01 mol (12 mL) of sunflower oil and 0.1 mol (6 mL) of absolute ethanol with free PPL (0.01 g) and immobilized PPL (0.04 g of lipase in 0.5 g of support), at different reaction times, different pH values and temperatures.

*N°*	*t* (h)	*T* (°C)	pH	FAEE (%)	MG + DG (%)	Yield (%)	Conv. (%)	TOF (mmol/h g_PPL_)
0 a	24	40	10	11.4	47.9	11.4	59.3	4.75
0 b	24	40	10	10.2	48.1	10.2	58.2	4.25
1	24	40	8	9.1	50.6	9.1	59.6	1.22
2	24	40	9	19.4	46.8	19.4	66.2	1.39
3	24	25	10	12.6	46.9	12.6	59.5	1.69
4	70	30	10	41.2	58.8	41.2	100	1.90
5	24	35	10	15.4	32.3	15.4	47.7	2.07
6	24	40	10	17.4	43.6	17.4	61.0	2.33
7	72	25	10	60.2	36.3	60.2	100	2.70
8	24	50	10	22.7	66.6	22.7	89.3	3.05
9	30	25	12	53.6	37.9	53.6	91.4	5.76
10	24	30	12	47.7	51.7	47.7	99.5	6.41
11	24	35	12	54.3	45.7	54.3	100	7.30
12	24	40	12	60.0	40.0	60.0	100	8.06
13	24	45	12	61.3	38.7	61.3	100	8.24
14	20	50	12	58.9	41.1	58.9	100	9.50

a Free enzyme (0.01 g);

b Free enzyme in the filtrate, alter the immobilization.

**Table 2:** Composition, yield, conversion and TOF of the biodiesel obtained in the transesterification reaction of sunflower oil with a different oil/ethanol ratio, with the same biocatalyst used in Table 1, 0.031 g of immobilized PPL (0.5 g support, 0.04 g lipase), working in standard conditions at pH = 12, 40 °C and different reaction times.

*N°*	Oil/Alcohol (mL/mL) (mol/mol)		Time (h)	FAEE (%)	MG + DG (%)	GLY (%)	Yield (%)	Conv. (%)	TOF (mmol/h g_PPL_)
15	24/3	0.020/0.05	27	69.1	30.80	0	69.1	100	16.51
16	21/3	0.018/0.05	24	55.9	44.1	0	55.9	100	13.52
17	18/3	0.015/0.05	20	45.5	54.6	0	45.5	100	11.01
18	15/3	0.013/0.05	20	57.0	43.0	0	57.0	100	11.95
19	12/3	0.01/0.05	20	49.9	50.1	0	49.9	100	8.04
20	12/4	0.01/0.07	20	53.4	46.2	0.5	53.6	100	8.64
21	12/6	0.01/0.01	18	57.5	35.6	6.9	61.8	100	11.08

**Table 3:** Composition, yield, conversion and TOF of the biodiesel obtained in the transesterification reaction with 0.01 mol (12 mL) of sunflower oil and 0.15 mol (6 mL) of methanol (MeOH), and the same biocatalyst used in Tables 1 and 2, 0.031 g of immobilized PPL (0.5 g support, 0.04 g of lipase), at different reaction times, different pH values and temperatures.

*N°*	*t* (h)	*T* (°C)	pH	FAME (%)	MG + DG (%)	GLY (%)	Yield. (%)	Conv. (%)	TOF (mmol/h g_PPL_)
22	24	25	12	59.5	40.5	0	59.5	100	8.00
23	24	30	12	61.3	38.7	0	61.3	100	8.24
24	25	35	12	62.5	31.5	6.1	66.5	100	8.64
25	24	40	12	60.8	30.5	8.7	66.6	100	8.95
26	23	45	12	57.8	28.9	13.3	66.7	100	9.36
27	30	25	10	55.7	36.0	8.4	60.7	100	6.52
28	30	30	10	60.0	30.0	10.1	66.7	100	7.17
29	26	35	10	60.4	33.7	5.8	64.2	100	7.96
30	24	40	10	60.5	32.8	6.7	64.8	100	8.72
31	24	45	10	60.3	30.2	9.5	66.6	100	8.96

**Table 4:** Composition, yield, conversion and TOF of the biodiesel obtained in the transesterificación reaction of 0.01 mol (12 mL) of sunflower oil with 0.08 mol. (6 mL) of 1-propanol and 2-propanol, with the same biocatalyst used before in Tables 1–3, 0.031 g of immobilized PPL (0.5 g support, 0.04 g of lipase), working in standard condition at pH = 10 and different temperatures and reaction times.

*N°*	Alcohol	*T* (°C)	*t* (h)	FAE (%)	MG + DG (%)	GLY (%)	Yield (%)	Conv. (%)	TOF (mmol/h g_PPL_)
32	1-PrOH	25	19	46.0	54.0		46.0	100	7.81
33	1-PrOH	30	22	55.7	39.6	4.7	58.4	100	8.57
34	1-PrOH	35	31	79.2	13.2	7.6	85.7	100	8.91
35	1-PrOH	40	28	80.9	17.0	2.0	82.6	100	9.52
36	1-PrOH	45	26	73.4	18.4	8.2	80.0	100	9.92
37	1-PrOH	50	19	58.3	35.8	5.9	62.0	100	10.52
38	2-PrOH	25	25	35.5	31.9	0	35.5	67.4	4.58
39	2-PrOH	30	24	34.3	35.1	0	34.3	69.4	4.61
40	2-PrOH	35	24	34.7	36.4	0	34.7	71.1	4.66
41	2-PrOH	40	23	33.9	55.6		33.9	89.5	4.75
42	2-PrOH	45	22	32.8	34.4	0	32.8	67.2	4.81
43	2-PrOH	50	23	35.5	29.8	0	35.5	65.3	4.98

**Table 5:** Effect of the different short-chain alcohols on composition, yield (as FAE, Fatty Acid Ester) and conversion (Conv., % by GC) and TOF (turn over frequency) of the Ecodiesel-100, obtained in the alcoholysis of sunflower oil (12 mL oil, 0.01 mol), 1/3 oil/alcohol molar ratio, 0.01 g free PPL (0.1% *w*/*w* of total substrate), 45 °C, pH = 12.

Alcohol	Time (h)	FAE (%)	MG + DG (%)	Yield (%)	Conv. (%)	TOF (mmol/h g_PPL_)
MeOH	24	55.1	44.9	55.1	100.0	22.9
EtOH	10	58.7	41.3	58.7	100.0	58.7
	24	60.7	39.3	60.7	100.0	25.5
EtOH 96%	10	27.8	72.2	27.8	100.0	27.8
	24	35.3	64.7	35.3	100.0	14.7
1-PrOH	16	56.9	43.1	56.9	100.0	35.6
	24	58.9	41.1	58.9	100.0	24.5
2-PrOH	16	19.6	80.4	19.6	100.0	12.3
	24	56.4	43.6	56.4	100.0	23.5
1-BuOH	16	47.5	42.2	47.5	89.7	29.7
	24	49.3	42.1	49.3	91.4	20.5
2-BuOH	13	59.6	40.4	59.6	100.0	45.8
	24	65.7	34.3	65.7	100.0	27.3
*t*-BuOH	24	52.3	38.3	52.3	100.0	21.8
1-PeOH	24	58.9	41.2	58.9	100.0	24.5

**Table 6:** Kinematic viscosity values, υ (cSt or mm^2^/s) at 40 °C of various representative biofuels obtained under homogeneous alkaline or enzymatic catalysis as well as under heterogeneous catalysis, using various alcohols. Kinematic viscosity of sunflower oil, commercial diesel and a B20 blend obtained from the worst biofuel (υ = 12.9 cSt) 20% and commercial diesel are also included.

Catalyst	Alcohol	FAE (%)	MG + DG (%)	Yield (%)	Conv. (%)	υo
NaOH	MeOH	95.7	4.3	95.7	100.0	3.9
KOH	EtOH	94.8	5.2	94.8	100.0	6.6
PPL free	EtOH	55.7	44.2	55.7	100.0	6.9
PPL immobilized	EtOH	61.3	38.7	61.3	100.0	4.1
PPL immobilized	1-PrOH	62.0	35.8	62.0	100.0	9.2
PPL immobilized	2-prOH	33.9	55.6	33.9	89.5	12.9
B20		-	-	-	-	4.2
Sunflower oil		-	-	-	-	31.9
Diesel		-	-	-	-	3.1

**Table 7:** Activation energy *E*_a_ (Kcal/mol) and Arrhenius pre-exponential factor Ln A (h^−1^), obtained in the transesterification reaction of 0.01 mol (12 mL) of sunflower oil with 6 mL of different alcohols using free PPL and the same biocatalyst as used before in Tables 1–4, 0.031 g of immobilized PPL (0.5 g support, 0.04 g of lipase).

Alcohol	pH	*E_a_* (Kcal/mol)	Ln A (h^−1^)	*r^2^*
Ethanol a	12	8.40 ± 0.24	17.76 ± 0.76	0.99
Ethanol	12	3.61 ± 0.15	7.87 ± 0.48	0.97
Ethanol	10	4.43 ± 0.13	7.99 ± 0.42	0.99
Methanol	12	1.43 ± 0.04	4.49 ± 0.13	0.99
Methanol	10	3.03 ± 0.15	7.01 ± 0.47	0.97
1-Propanol	10	2.16 ± 0.05	5.71 ± 0.17	0.99
2-Propanol	10	0.60 ± 0.05	2.52 ± 0.16	0.90

a Free PPL (0.01 g), data are from a previous paper [37].
